# Supplementary material for: Changes in physiotherapists’ perceptions of evidence-based practice after a year in the workforce: A mixed-methods study
Source: PLoS One. 2020 Dec 21;15(12):e0244190. doi: 10.1371/journal.pone.0244190 (PMC7751960; doi:10.1371/journal.pone.0244190)
Supplement: S1 Appendix — (DOCX) [file pone.0244190.s001.docx]

**Semi-structured interview question guide**

**(General experience)**

First, I’d like to ask you about how, in general, it’s been as a new graduate in the workplace.

Could you tell me about the places/areas, you have worked over the last 9 months and the kinds of patients you have been involved with?

In general, how have you found your first year in the workplace?

How would you describe your workplace in relation to general support and encouragement for a new graduate?

**(Experience of EBP in workplace)**

In relation to EBP, do you feel EBP is ‘valued’ in your workplace? ...by managers/supervisors, …by peers? Can you give me examples to help me understand?

Have your EBP skills and knowledge been relevant during this first year in the workplace?

Have you used your EBP skills and knowledge during your first year in the workplace? Can you give me any examples to help me understand?

Please tell me about any opportunities you have had to put EBP into practice during your first year in the workplace…maybe a few examples?

If you have been able to put EBP into practice, do you think it influenced your patient management/decision-making? Can you tell me a bit more about this?

Do you think your confidence in EBP skills and knowledge has increased, decreased or stayed the same over the first year of work? Could you give me examples to help me understand?

**(Patients’ perceptions of EBP)**

Do you think patients you have seen in the workplace have a view about EBP? Can you give me any examples to help me understand?

**(Role-models for EBP)**

Without naming anybody, in your workplace over the past year, is there anyone that you can think of that you would consider a role model for EBP?

Can you describe the kinds of behaviours that this role model demonstrates…or can you describe, what kinds of behaviours or attributes make this person a role model?

OK, thanks for that. I think I understand why this person is a Positive/Negative role model for EBP. I wonder whether you can think about anyone in your workplace who might be the reverse, that is a Positive/Negative role model for EBP…

Can you describe the kinds of behaviours that this role model demonstrates…or can you think about and describe, what kinds of behaviours or attributes make this person a role model?

Do you think that overall, your workplace demonstrates an EBP approach to patient management?

If yes - Can you tell me why or what sorts of things encourage the use of EBP in your workplace? What do you think works well?

If no - can you tell me why or what sorts of things discourage the use of EBP in your workplace? What do you think would help encourage EBP in your workplace?

**(Reflecting on EBP training)**

Having worked now for a period, I’m interested to hear your thoughts/reflections about the training your received in EBP as a student. Overall do you think your undergraduate training in EBP prepared you for the workplace?

If yes - What worked well? What could be improved?

If no - What didn’t work?

What would you change in the EBP undergraduate training?
