# Supplementary material for: Changes in physiotherapists’ perceptions of evidence-based practice after a year in the workforce: A mixed-methods study
Source: PLoS One. 2020 Dec 21;15(12):e0244190. doi: 10.1371/journal.pone.0244190 (PMC7751960; doi:10.1371/journal.pone.0244190)
Supplement: S2 Appendix — (PDF) [file pone.0244190.s002.pdf]

| Participant | Timepoint | Age | Gender | T1relevanc | T1sympath | T1practice | T1Confider | T1termino | T1KREC |
|---|---|---|---|---|---|---|---|---|---|
| 1 | 1 | 21 | 1 | 52 | 16 | 18 | 39 | 52 | 9 |
| 2 | 1 | 23 | 1 | 70 | 26 | 36 | 55 | 85 | 9.5 |
| 3 | 1 | 21 | 1 | 65 | 27 | 29 | 44 | 70 | 11.5 |
| 4 | 1 | 21 | 2 | 69 | 29 | 35 | 52 | 81 | 7.5 |
| 5 | 1 | 21 | 2 | 58 | 20 | 29 | 47 | 75 | 7 |
| 6 | 1 | 22 | 1 | 62 | 24 | 33 | 47 | 68 | 9.5 |
| 7 | 1 | 22 | 2 | 69 | 29 | 25 | 45 | 63 | 8.0 |
| 8 | 1 | 20 | 1 | 67 | 24 | 21 | 42 | 71 | 8.5 |
| 9 | 1 | 22 | 1 | 63 | 26 | 28 | 42 | 70 | 10.5 |
| 10 | 1 | 21 | 1 | 68 | 26 | 18 | 49 | 60 | 7.5 |
| 11 | 1 | 21 | 1 | 65 | 21 | 21 | 36 | 48 | 11 |
| 12 | 1 | 21 | 1 | 70 | 25 | 25 | 47 | 81 | 8 |
| 13 | 1 | 20 | 1 | 66 | 27 | 28 | 49 | 77 | 9 |
| 14 | 1 | 22 | 1 | 55 | 25 | 28 | 48 | 62 | 9.75 |
| 15 | 1 | 21 | 2 | 57 | 22 | 23 | 31 | 55 | 8.5 |
| 16 | 1 | 23 | 1 | 69 | 31 | 19 | 46 | 68 | 9.5 |
| 17 | 1 | 21 | 1 | 57 | 23 | 21 | 41 | 64 | 9.5 |
| 18 | 1 | 20 | 1 | 57 | 23 | 18 | 35 | 63 | 9.5 |
| 19 | 1 | 21 | 1 | 65 | 24 | 27 | 50 | 75 | 8 |
| 20 | 1 | 21 | 2 | 62 | 23 | 25 | 43 | 81 | 8 |
| 21 | 1 | 21 | 2 | 65 | 24 | 20 | 49 | 70 | 8.5 |
| 22 | 1 | 23 | 1 | 68 | 23 | 28 | 55 | 68 | 9 |
| 23 | 1 | 23 | 2 | 63 | 26 | 32 | 40 | 54 | 7 |
| 24 | 1 | 22 | 1 | 67 | 22 | 23 | 40 | 52 | 8 |
| 25 | 1 | 22 | 1 | 63 | 17 | 37 | 36 | 65 | 11.0 |
| 26 | 1 | 28 | 1 | 63 | 23 | 33 | 51 | 67 | 9.5 |
| 27 | 1 | 23 | 2 | 69 | 25 | 27 | 39 | 52 | 8.5 |
| 28 | 1 | 22 | 1 | 68 | 27 | 23 | 42 | 69 | 9.5 |
| 29 | 1 | 22 | 1 | 66 | 18 | 27 | 38 | 60 | 8 |
| 30 | 1 | 22 | 2 | 64 | 24 | 32 | 51 | 68 | 9 |
| 31 | 1 | 21 | 1 | 70 | 25 | 34 | 49 | 68 | 8 |
| 32 | 1 | 21 | 2 | 70 | 30 | 26 | 43 | 66 | 0 |
| 33 | 1 | 47 | 1 | 70 | 25 | 36 | 49 | 81 | 8.5 |
| 34 | 1 | 29 | 1 | 63 | 19 | 20 | 40 | 62 | 11.5 |
| 35 | 1 | 21 | 2 | 63 | 26 | 31 | 49 | 74 | 8.5 |
| 36 | 1 | 22 | 1 | 69 | 26 | 18 | 46 | 65 | 9 |
| 37 | 1 | 22 | 1 | 61 | 15 | 25 | 48 | 72 | 8 |
| 38 | 1 | 22 | 1 | 67 | 25 | 31 | 47 | 74 | 9 |
| 39 | 1 | 21 | 1 | 70 | 27 | 29 | 50 | 76 | 10 |
| 40 | 1 | 22 | 1 | 65 | 26 | 25 | 46 | 67 | 8 |
| 41 | 1 | 21 | 1 | 68 | 23 | 34 | 48 | 76 | 8.5 |
| 42 | 1 | 21 | 1 | 65 | 20 | 26 | 51 | 75 | 8 |
| 43 | 1 | 23 | 2 | 64 | 25 | 33 | 45 | 50 | 9 |
| 44 | 1 | 21 | 2 | 61 | 20 | 21 | 42 | 78 | 9.5 |
| 45 | 1 | 23 | 2 | 70 | 28 | 26 | 44 | 64 | 10 |
| 46 | 1 | 22 | 1 | 70 | 20 | 36 | 55 | 79 | 11.5 |
| 47 | 1 | 21 | 1 | 64 | 24 | 18 | 36 | 57 | 12 |
| 48 | 1 | 21 | 1 | 66 | 26 | 30 | 28 | 53 | 11 |
| 49 | 1 | 23 | 1 | 65 | 29 | 28 | 48 | 53 | 10 |

| | | | | | | | | | |
|---|---|---|---|---|---|---|---|---|---|
| 50 | 1 | 23 | 2 | 65 | 25 | 31 | 42 | 66 | 8.5 |

| Timepoint 2 | T2relevance | T2sympathy | T2practice | T2Confidence | T2terminology | T2 KREC |
|---|---|---|---|---|---|---|
| 2 | 60 | 24 | 21 | 30 | 56 | 9.5 |
| 2 | 68 | 22 | 34 | 52 | #NULL! | 7 |
| 2 | 60 | 20 | 34 | 49 | 78 | 11.5 |
| 2 | 65 | 30 | 40 | 51 | 82 | 11 |
| 2 | 66 | 26 | 35 | 52 | 77 | 11 |
| 2 | 45 | 20 | 21 | 37 | 52 | 9 |
| 2 | 64 | 29 | 24 | 45 | 60 | 9 |
| 2 | 55 | 22 | 20 | 31 | 68 | 9 |
| 2 | 57 | 26 | 25 | 33 | 59 | 7 |
| 2 | 69 | 24 | 22 | 36 | 45 | 9 |
| 2 | 62 | 22 | 21 | 31 | 50 | 9.5 |
| 2 | 61 | #NULL! | 25 | 45 | 83 | 8 |
| 2 | 63 | 21 | 27 | 48 | 66 | 6.5 |
| 2 | 61 | 23 | 22 | 40 | 61 | 8.5 |
| 2 | 49 | 20 | 19 | 24 | 48 | 7.5 |
| 2 | 62 | 24 | 26 | 48 | 76 | 9 |
| 2 | 57 | 25 | 25 | 40 | 57 | 9 |
| 2 | 58 | 23 | 17 | 42 | 74 | 9.5 |
| 2 | 59 | 21 | 31 | 40 | 67 | 7.5 |
| 2 | 57 | 18 | 26 | 40 | 71 | #NULL! |
| 2 | 60 | 22 | 22 | #NULL! | 56 | 9.5 |
| 2 | 64 | 25 | 31 | 44 | 68 | 8 |
| 2 | 59 | 24 | 26 | 40 | 60 | 8 |
| 2 | 57 | 21 | 22 | 31 | 50 | 9.5 |
| 2 | 64 | 25 | 29 | 40 | 84 | 9 |
| 2 | 53 | 22 | 16 | 40 | 61 | 8 |
| 2 | 67 | 24 | 24 | 43 | 46 | 9 |
| 2 | 57 | 27 | 21 | 43 | 72 | 8.5 |
| 2 | 59 | 20 | 24 | 39 | 62 | 6.5 |
| 2 | 69 | 26 | 29 | 55 | 77 | 9 |
| 2 | 63 | 22 | 24 | 50 | 73 | 8 |
| 2 | 69 | 29 | 37 | 35 | 59 | 9 |
| 2 | 62 | 28 | 25 | 52 | 79 | 8 |
| 2 | 53 | 23 | 18 | 34 | 58 | 10 |
| 2 | 69 | 28 | 34 | 47 | 57 | 7.5 |
| 2 | 63 | 22 | 29 | 38 | 52 | 10 |
| 2 | 67 | 19 | 28 | 48 | 76 | 7 |
| 2 | #NULL! | 25 | 27 | 52 | 79 | 9.5 |
| 2 | 64 | 29 | 23 | 42 | 72 | 11.5 |
| 2 | 59 | 21 | 20 | 31 | 56 | 7.5 |
| 2 | 51 | 19 | 18 | 33 | 51 | 6.5 |
| 2 | 67 | 27 | 36 | 47 | 78 | 9 |
| 2 | 58 | 27 | 18 | 42 | 46 | 9 |
| 2 | 66 | 25 | 27 | 41 | 73 | 8.5 |
| 2 | 66 | #NULL! | 23 | 44 | 71 | 8.5 |
| 2 | 63 | 22 | 26 | 40 | 66 | 9 |
| 2 | 64 | 27 | 18 | 23 | 60 | 10 |
| 2 | 60 | 21 | 30 | 40 | 52 | 9.5 |
| 2 | 56 | 26 | 25 | 39 | 61 | 7 |

| | | | | | | |
|---|---|---|---|---|---|---|
| 2 | 55 | 21 | 28 | 38 | 63 | 8.5 |
