# Supplementary material for: Changes in physiotherapists’ perceptions of evidence-based practice after a year in the workforce: A mixed-methods study
Source: PLoS One. 2020 Dec 21;15(12):e0244190. doi: 10.1371/journal.pone.0244190 (PMC7751960; doi:10.1371/journal.pone.0244190)
Supplement: Attachment — Submitted filename: Response to Reviewers.docx [file pone.0244190.s004.docx]

| **RESPONSE TO REVIEWERS** | *All page/line numbers refer to those in the marked-up copy |
| --- | --- |
| **‘Changes in physiotherapists perceptions of evidence-based practice after a year in the workforce: a mixed-methods study’** |  |
| Additional changes from the authors | Additional minor changes to the Abstract have been made to reflect changes in the manuscript  Please note a change in the name of the school for the first three authors (1) within the University of South Australia from ‘School of Health Sciences’ to ‘Allied Health and Human Performance Unit’ |
| **Reviewer #1 OVERALL** | Thank you for your considered review of our paper. We have responded in the table below to each point you have raised. |
| 1.The contribution to knowledge gap of this research report is undeniable. There are hardly any studies on evidence uptake after having learned the principles of EBP in entry-level education particularly in Physiotherapy. Therefore it is important that the findings of this research be communicated with clarity so that readers and follow through the data collection procedures, data analysis and insights of the authors. | Yes, we agree that this is a gap.  Thank you for your review and we hope the amendments have improved the clarity of the manuscript. |
| 2.Background/introduction. The objective depicts a qualitative study when the entire paper talks about mixed- methods. Provide an objective that would justify quantitative data collection and process. | The background has been re-structured to clarify and justify the aims of the study.  Page 4, lines 64-69 previously read:  “Despite this, while McEvoy et al. [13] provided qualitative data on the workplace transition, we currently have not explores further what happens to EBP knowledge, attitudes and behaviour in the physiotherapy workplace after graduation.”  This section now reads:  “McEvoy et al. [13] also provided quantitative data on changes in EBP knowledge, attitudes and behaviour after transitioning into the workplace for one and two years. However, there was no further exploration of what may influence these changes.”  Page 5, lines 79-80, previously read:  “Therefore, this study aimed to explore changes in EBP knowledge, attitudes and behaviours in a cohort of physiotherapists, from graduation to one year in the workforce.”  This now reads, Pg 5, lines 82-86:  “Therefore, the objective of this study was to determine what is influencing the use of EBP by physiotherapists during their first year in the workforce. The specific aims of the study were to quantitatively examine overall changes in domain scores associated with EBP knowledge, attitudes and behaviours, and to qualitatively explore the experience of using EBP in a cohort of physiotherapists after a year in the workforce.” |
| 3.Design. Explain more clearly how qualitative and quantitative procedures were used for the study. Was one dependent on the other? If qualitative data was used to support quantitative data, how and when was the qualitative arm of the study done? Did the authors analyzed the quantitative findings in order to direct or focus their interview questions? Or did they ask the interview questions independent of the quantitative results? | This point was also raised by the other reviewer and highlighted our lack of detail about the Mixed Methods design used in our study. We have now added information and a reference to address this.  Page 5/6, lines 94-99 previously read,  “This mixed-methods study used quantitative and qualitative methods to collect information from participants. Quantitative data were collected with two questionnaires at two timepoints (graduation and after one year in the workforce). Qualitative data were collected with individual interviews at one timepoint (after one year in the workforce) to gain a deeper and broader understanding of graduates’ first year of work and EBP experience. “  This now reads, page 6, lines 104-113:  “Quantitative and qualitative procedures of the study were conducted independently of each other in a convergent mixed methods design [17]. The timepoint 1 quantitative data was collected on participants’ completion of the final EBP course in their final year of the Physiotherapy Program. The timepoint 2 quantitative data was collected after participants had been in the workforce for up to 1 year (September-November 2014). The qualitative interviews were conducted in the immediate period after the timepoint 2 quantitative data was collected (November 2014). The interview questions were independent of the quantitative results and not all participants who completed the survey, consented to the interviews. The quantitative and qualitative data were analysed separately then merged in the interpretation and reporting of results.”  Two sentences have been added to the ‘Data management and analysis’ section to report the method of data integration used. It now reads on page 10, lines 218-221,“The quantitative and qualitative findings of the study were integrated narratively to address the study objective [17].The quantitative findings are presented first then the subsequent reporting of qualitative findings uses a weaving approach to integrate the qualitative themes with the quantitative domain results.” |
| 4.Description of participants; who was the target population, how many were they? What are the inclusion and exclusion criteria? | Further detail about the population and inclusion and exclusion criteria have been added.  Page 6, line 110-114 previously read:  **“Participants**  “Participants were physiotherapists who completed their final year of entry-level training at the University of South Australia in 2013 and graduated and entered the physiotherapy workforce in 2014.”  This now reads, page 7, lines 125-130  **“Participants**  The target population was all physiotherapy students completing their final year of training at the University of South Australia in 2013 and entering the physiotherapy workforce in 2014 (n=125). For inclusion, students needed to have completed all final year courses in late 2013 or early 2014, and to be entering the physiotherapy workforce (i.e. not continuing to further study, not delaying practicing as a physiotherapist). International students leaving Australia were excluded.” |
| 5.How was quantitative data treated? The authors mentioned that only respondents who answered both sets of questionnaires in Times 1 and 2 were included. Yet there are respondents who did not answer all items as expressed in lines 182-184? ‘there was not complete matched data from all participants for each of the six EBP domains’? | In the development and psychometric testing of the Evidence Based Practice Profile questionnaire, principle component factor analysis was used, and five factors were identified. These different factors or domains of EBP allowed for analysis on each domain independently. For inclusion of data in the analysis of this questionnaire, a participant needed to complete ALL items within a domain, but may not have completed all items in the whole questionnaire. For example, for some participants they may have scores for 4 or 5 of the domains of the Evidence Based Practice Profile Questionnaire. The sixth domain was actual knowledge’, based on the Knowledge of Research Evidence Competencies (KREC) questionnaire.  This has been clarified in Data, management and analysis.  Page 7, lines 148-150 previously read  “Participants were only included if matched data were available for the two timepoints.”  This now reads, page 9, lines 187-189:  “Participants’ scores for the domains were only included if matched data for the domain were available for the two timepoints. If all items in a domain on either occasion were not completed, this domain score was not included in the analysis.” |
| 6.The authors stated that a limitation of this study is the method of analysis for the qualitative data. It puts into question the results of the study; why wasn’t an “in-depth” analysis conducted when that was the intention for doing a qualitative arm as stated in the design? This has to be clarified in order to align methodology and results. | Thank you for raising this point. We agree that identifying our choice of descriptive thematic analysis as a limitation is erroneous and may unintentionally question the results of our study. The sentence has been removed.  The aim of the qualitative aim of the study is now clearly stated on page 5, lines 86-87, as “to qualitatively explore the experience of using EBP in a cohort of physiotherapists after a year in the workforce.” The justification for use of a descriptive thematic approach is provided on page 6, lines 117-119. |
| 7.Discussion. Most of the concepts were not followed through and requires further explanation and insights from the authors. This is very important if the results of the studies are to be useful for readers. | Thank you. In line with other comments made by the reviewers, the discussion has been expanded and greater depth has been provided, to give further explanation and the insights of the authors. |
| *8.Minor to moderate concerns are the following:* Sentence and paragraph construction could be greatly improved for readability and clarity. | The authors have paid particular attention to the readability and clarity of the manuscript and modified sentence and paragraph construction throughout. |
| 9.Formatting of tables for ease of reading the results and findings. | The authors have paid particular attention to reformatting tables in the results to enhance clarity |
| 10.I gave some suggestions that could be useful but authors are free to work around this. | Thank you for such detailed and thoughtful feedback and the time that this will have taken you. |
| 11.Addressing these concerns could make this manuscript more acceptable. Please find below specifics of my general review: | Thank you |
| 12.Please find below specifics of my general review: ABSTRACT Line 22. May I suggest to use physiotherapy participants or simply participants, instead of physiotherapy students since they were not students at the time of the study. | ‘physiotherapy students’ has been changed to ‘participants’ where appropriate |
| 13.BACKGROUND Lines 79 to 80. I suggest to include an aim or objective that would pertain to the quantitative aspect of the research in order to anticipate the collection of quantitative data. Eg. To determine changes in EBP profile… | The aim/objective has been clarified to address both quantitative and qualitative aspects of the research:  Page 5, lines 79-80, previously read:  “Therefore, this study aimed to explore changes in EBP knowledge, attitudes and behaviours in a cohort of physiotherapists, from graduation to one year in the workforce.”  This now reads, page 5, lines 83-87:  “Therefore, the objective of this study was to determine what is influencing the use of EBP by physiotherapists during their first year in the workforce. The specific aims of the study were to quantitatively examine overall changes domain scores associated with EBP knowledge, attitudes and behaviours, and to qualitatively explore the experience of using EBP in a cohort of physiotherapists after a year in the workforce.” |
| METHODS 14.Design Line 94, remove period after workforce since the sentence is not yet completed. | Thank you. The period has been removed. |
| 15.How did the researchers treat the two methods for data collection? Was one dependent on the other or were they treated independent of each other. Were the interviews conducted taking into consideration the changes in scores of the quantitative data? | Please see our response to point 3. |
| 16. Participants Line 106, Is it possible that one can complete entry-level of training in university and not graduate? If completion of entry-level training subsequently leads to being ‘graduated' then you can delete the words ‘and graduated’ to go straight to ‘and entered the physiotherapy workforce…’. | Thank you. The words ‘and graduated’ have been removed. |
| 17.Are there exclusion criteria for the participants? (I think you mentioned these in the results part. You can mention it instead here). | Thank you. Please see our response to point 4. |
| 18.Lines 107 - 108. Consider revising the sentence, starting with your intention to achieve a 80% power. Example. In order to achieve a power of 80% for the quantitative data……a minimum of 34 participants were needed. Or: A minimum number of 34 participants was needed for the quantitative data set in order to obtain an 80% power for a two-tailed distribution, with alpha set at 0.05 and a medium effect size (d=0.5). | The sentence (page 7, lines 131-133) has been revised: “A minimum number of 34 participants was needed for the quantitative data set in order to obtain 80% power for a two-tailed distribution, with alpha set at 0.05 and a medium effect size (d=0.5) derived from the study of McEvoy et al [13].” |
| Procedure  19. Line 112. Consider improving the sentence construction in describing the questionnaires for easier reading and comprehension. Example: Quantitative data were collected using two questions, the EBP2 and the KREC. The EBP2 has been psychometrically tested and shown to be valid and reliable etc.  It may not be necessary to state that it is developed, since it has been used several times already. Then move on to describe its features; number of items, domains etc. | The sentence construction in describing the two questionnaires has been improved for clarity. Summary information about validity and reliability has been added as the authors have interpreted the reviewers to be asking for this additional validity and reliability information. This now reads, pages 7-8, lines 141-155:  “The EBP^2^ questionnaire has been psychometrically-tested [21] and shown to be valid and reliable (test-retest reliability: ICC 0.77 to 0.94, internal consistency: Cronbach’s alpha 0.96, convergent validity: Pearson r= 0.54-0.80, can distinguish between groups for different levels of EBP exposure p<0.05) The EBP^2^ questionnaire includes 58 items (5-point Likert scale) that relate to five self-reported EBP domains: *Relevance* (14 items) is the value, emphasis or importance placed on EBP; *Terminology* (17 items) relates to an understanding of common research terms; *Confidence* (11 items) is perception of ability in EBP skills; *Practice* (9 items) is the application of EBP in clinical decision-making; and *Sympathy* (7 items) is the perceptions of compatibility of EBP with day-to-day professional work [21]. The K-REC questionnaire has 9-items that measure participants’ knowledge of EBP using multiple choice, true and false and an open-ended question, pertaining to a clinical scenario, and has been demonstrated to be a valid and reliable tool. K-REC questionnaire has been found to be valid and reliable (test-retest reliability: Cohen’s Kappa and ICC range 0.62 to perfect agreement, can distinguish between groups with and without formal EBP training p<0.0001) [22].” |
| 20.Line 119. Is 'actual knowledge’ used as a proper noun here? also why is it italicized? When using the word "subsequent", do you mean it was answered AFTER the EBP2? Was this an important or significant step in the process? Would it have mattered if participants answered K-REC first before EBP2? If the sequence in which the questionnaires were answered does not matter, then no need to write the word ‘subsequent'. If the sequence mattered, kindly give a brief explanation for this. The K-REC questionnaire has nine items that measures a participants knowledge of EBP using… It has been found to be valid and reliable….etc. | Thank you: the italics have been removed from 'actual knowledge’ in this sentence.  Thank you: the word ‘subsequent’ is mis-leading, and has been removed.  The sentence construction in describing the K-REC has been improved for clarity and is presented in our response to comment 19. |
| 21. Line 123. Use an active voice when possible. Example: Participants completed the questionnaires at two timepoints; 1) upon their completion of entry-level training, Time 1, and 2) one year after entering the workforce, Time 2. At time point 1, participants answered the questionnaires using a pen-and-paper format. For time point 2, participants answered using an electronic version of the questionnaire through Survey Monkey, due to the participants' being geographically dispersed  Suggestion. How about using Time 1 and Time 2 instead of Timepoint 1, Timepoint 2.? If you agree, please revise throughout the manuscript. | Thank you; the active voice has been used.  This now reads for lines 157-163:  “Participants completed the questionnaires at two timepoints: 1) at completion of entry-level training, Timepoint 1, and 2) one year after entering the workforce, Timepoint 2. At Timepoint 1, participants answered the questionnaires using a pen-and-paper format. For Timepoint 2, participants answered using an electronic version of the questionnaires through Survey Monkey, due to the participants being geographically dispersed.”  The authors used ‘Timepoint’ rather than ‘Time’ to avoid any confusion with use of the word ’time’ for a) ‘occasions’ and more importantly for b) ‘sufficient hours or minute to undertake the EBP process’ which was a common barrier to practice of EBP reported and discussed in this manuscript.  The authors would like to continue with the use of the word ‘Timepoint’. |
| 22.Lines 131 to 133. When you say that “feedback from a reference group of six ….”, were you talking about content validation of the interview guide or did you pilot test the interview guide with this reference group? Also, my downloaded copy of the manuscript did not include S1 Appendix being referred to at this part. | The sentence has been revised for greater clarification of feedback purpose.  Page 8, lines 154-157 previously read,  “A semi-structured interview guide was developed by the research team with feedback from a reference group of six physiotherapists with broad EBP teaching experience, including a researcher in this field (S1 Appendix).”  This now reads, lines 170-172:  “Content validity of the interview guide was supported by seeking feedback during its development from a reference group of six physiotherapists with broad EBP teaching experience, including a researcher in this field (S1 Appendix).”  The semi-structured interview question guide was submitted as an appendix in the initial manuscript submission. |
| 23.Line 136. Under Design, I commented/recommended that you state the relationship of the two methods of data collection. It might be better to place lines 136 - 138 in that area of the paper. So that this part of the paper, similar to quantitative data collection, will discuss data collection tools and data collection process. So instead, you can proceed by describing the interview guide in terms of the topics covered. | We agree with your suggestion about location of this information reporting the aim of the qualitative arm.  Line 136 has been removed from the Methods Procedure section.  The content of line 136 has been integrated into our revisions to the Design section. Please see our response to point 3. |
| 24.Line 141 onwards. An interviewer, independent of the research team and experienced in qualitative interviewing, conducted telephone interviews with the participants | Thank you. Lines 178-179 now read:  “An interviewer, independent of the research team and experienced in qualitative interviewing, conducted telephone interviews with the participants.” |
| DATA MANAGEMENT AND ANALYSIS 25.Line 148 onwards. Suggestion: The predictive Analytic Software (PASW) Statistics 17.0 was used for quantitative data analysis. Only completed matched data (timepoints 1 and 2) were included for analysis. Consider breaking down sentences when conveying different ideas or thoughts. Eg. Separate the sentences when talking about Likert scores and the fact that the domains have different number of items. Example: The Likert-scores for the EBP2 were treated as interval data. Then move on to describe the domain score. Is there something more that you would want to discuss in reference to the different domain scores? Did this matter in data analysis. Because if this sentence (lines 150-152) just means to say that the scores will be different, it can be derived from the earlier description of the instrument, wherein the number of items for each domain was listed. But if this was transformed to make them ‘equal' for data analysis, then maybe this should be described. | The sentences have been re-structured for greater clarity and to separate thoughts and ideas.  The sentence in lines 189-190 now reads “The Likert scores for the EBP^2^ questionnaire were treated as interval data.”  The different number of items and therefore domain scores needed to be considered when presenting the domains on a single figure in the Results. To allow this to be done, the domain scores as a percentage of possible maximum (100%) were calculated.  This process was previously included in the Results section, but will now be moved to the quantitative analysis section.  In lines 197-199 a sentence has been added: “As maximum domain scores varied due to the different numbers of items, the scores were calculated as a percentage of possible maximum (100%) at each timepoint, for graphical presentation.” |
| 26.Line152. I don’t think it is necessary to include ‘for Actual Knowledge' to this. The K-REC gives a maximum score of 12. The participants responses were sacred using the set scoring guidelines. | Thank you. The phrase ‘for Actual Knowledge' has been removed and the sentence line 191-17892 now reads:  “The K-REC instrument gives a maximum score of 12. Participant responses were scored using set scoring guidelines.” |
| 27.Line 154. delete 'Actual Knowledge domain’ | The words 'Actual Knowledge domain’ have been removed. |
| 28.Line 160 to 168. I think this paragraph can be refined by using an active voice; state clearly what steps were taken for analysis; then towards the end escribe the entire process as iterative so that it does not have to be stated repeatedly in the paragraph.  Two researchers (CF and JL) independently (or separately) analyzed the qualitative data using a staged process of thematic analysis. First researchers read the transcripts repeatedly to familiarize themselves with the data. Second, codes were inductively allocated to small sections. Third, comparison across transcripts and codes were done, inductively grouping the codes in a meaningful manner to form categories and themes within each category. This was an iterative process requiring the researchers to meet and discuss regularly to eventually reach a consensus | Thank you. The paragraph, (lines 209-215) has been amended as suggested. |
| 29.In reference to line 163: Do the researchers think that analysis using software and hard copies affected the data? If so, maybe a short explanation is warranted. If not, this then can be deleted. | As the authors do not consider that analysis using software and hard copies affected the data.  The phrase: ‘’CF working on NVivo and JL on hard copies‘’ has been deleted |
| RESULTS QUANTITATIVE 30.May I suggest to use 'completed an entry-level physiotherapy program’ as descriptors for participants to veer away from their ‘student’ status. This would help in focusing the readers, that the study is following through working physios instead of students. | Thank you making this observation. We agree that this keep the focus on the transition through to working physiotherapists. We will make these changes throughout. |
| 31.The count of the participants is hard to follow through but it would be helpful if you keep in mind your target participants (physios who competed their final year of entry-level training at USA in 2013). So start with this number (how many were they actually). From this number, subtract those that copy with your exclusion criteria (plan to work overseas, not work in physio and did not consent to participate after one year). I think you will end up with 65 eligible participants. Then continue to discuss what happened to these eligible participants and how many viable data were left. Coming up with an illustration or figure for this might be helpful for the reader to follow. | As summarised in the first paragraph of the methods, lines 91-92, the current study followed on from a study evaluating changes from first year to graduation after a suite of EBP courses across the physiotherapy program. Some of the participants completing Timepoint 1 data for the current study may have completed final EBP courses but may not have completed all courses in the program to be graduating.  A figure, (Fig 1) has been added to illustrate the flow of participants through the study.    The associated text now reads, in lines 225-234:  “Fig 1 illustrates the flow of participants through the study. Of the 125 eligible participants, 87 had complete questionnaire data at timepoint 1 [12]. Of these, 84 participants consented for follow-up questionnaires at timepoint 2 after one year in the workforce, but 19 were ineligible for follow-up (failed to successfully complete final courses in the physiotherapy program, planning to work overseas or not to work in physiotherapy in the first year after graduation), resulting in 65 eligible participants for timepoint 2. Of these, 50 (70%) participants completed the questionnaires at timepoint 2. Of these 50 participants, 14 participated in interviews after timepoint 2 completion of questionnaires.” |
| 32.At this point, I am confused as to how many viable data were analyzed? What do the researchers mean by lines 182-184? ‘there was not complete matched data from all participants for each of the six EBP domains’? how then was this treated? Best also to describe participants at timepoints 1 and time point 2. At Time 1, 50 participants were mostly female with a mean age of 22 +/- 4 (range 20 - 47 years) At Time 2, 14 participants were interviewed, there were 10 females, with an average of 22 +/- 1 year (range 20 to 23). I think it is important to give characterization as to their workplace or work setting. This could give a better context of the succeeding discussion of results. | Please see our response to point 5 for clarification of matched data.  Participants who completed questionnaires will be described at timepoint 1 (and were a year older at timepoint 2). The participants involved in the interviews at timepoint 2 will described, including some information about their workplace or work setting.  Lines 238-244 now read:  “At timepoint 1, of the 50 participants there were 35 females and 15 males. The mean age was 22 +/- 4 (range 20 - 47 years). At timepoint 2, 14 participants were interviewed. There were 10 females and 4 males, with an average of 22 +/- 1 year (range 20 to 23). The interviewees worked in a range of settings in hospitals (n=4), private practice (n=7) and community health (n=3), in both metropolitan (n=9) and regional (n=5) areas. Most interviewees treated both inpatients and outpatients ± aged care (n=7), while others treated outpatients only ± sports (n=5) and inpatients only (n=2).” |
| 33.Table 1 can be further improved for easy reading. Separate the cells for Change “findings”; one cell each for raw score difference, p-value and effect size. You can also put as footer for the table, the meaning of Time 1 (completion of entry-level training) and Time 2 (one year working?). The title of table can also be improved. It should be able to answer the following; what is being presented? how is the data classified? Indicate number of data/participants. It might help if you look at other tables | Thank you, Table 1 has been modified in accordance with the reviewer’s suggestions. |
| 34.Figure 1 is a good illustration to show the change. It could be more informative if the data re: ES, p-values be shown there as well. Eg. Relevance (p-<.001, ES = .69) | Fig 1 (now Fig 2) has been modified with addition of p values and ES (see Fig 2 at the end of this document) |
| 35.Lines 207-209. Elaborate and describe a bit more what happened to the maximum domain scores. i believe this could be better situated and described under data management and analysis. | This has been added to the data management and analysis section as previously described in point 25 |
| QUALITATIVE 36.I think the results should answer the aim or objective of the research as indicated in the background; "To explore the changes in EBP knowledge, attitudes and behaviors from graduation to one year in workforce of a cohort of physiotherapists." Therefore, just present the themes gathered that best represents this aim. It is unnecessary to discuss how the other categories will be treated if it has nothing to do with the current paper. I think this adds to confusion and questions regarding the paper. I would suggest further improving your table that shows qualitative findings. Tables are meant for the reader to see at a glance the most significant findings of a study. Having 12 pages of this makes it difficult to do that. My suggestion…include samples of quotations from the respondents in the text instead of put them on the table. For the table, you may reformat to just include the themes and sub-themes. Example: Example in text: Most participants reported using EBP skills and knowledge in their first year to inform their clinical reasoning, empower clients with knowledge or learn from the clinical expertise of experienced colleagues. Some example to attest to these are: "So I’ve seen a few patients that I was having trouble with, searched some evidence, had a look at some new treatment techniques and stuff like that and obviously searched if they were significantly… sort of if they were going to work and tried them clinically and they did work, which was very helpful." (Participant 14 for informing clinical reasoning)  "Look I’ve had a few patients that they may have been a little bit apprehensive about some of the treatments that we use but then we sort of explain to them about the evidences behind it and it really does help them to feel confident in what we’re doing and that we’re not just doing some sort of weird treatment, that we actually have got evidence behind it and we’ve done research into it." (Participant 56, Empowering patients)  When using this format, the reader is easily referred to sample quotations without having to go back to the table.  This is a suggestion. There may be other ways to improve this. The main goal is to make the table an easy reference point. | Thank you for your feedback on presentation of the qualitative findings. The points raised by both reviewers prompted us to return to the analysis for further iterative work across codes and categories to define themes that are meaningful to the qualitative aim of how physiotherapists experience using EBP in their first year in the workforce. A Braun and Clarke (2012) reference [24] informing this thematic analysis process has been added to the Methods ‘Data management and analysis’ section.  The reporting of the qualitative results has been revised from page 14 and uses narrative reporting of seven key and meaningful themes with illustrative quotes only in the text as suggested. The seven key themes describe the key findings of the qualitative analysis, which had already been highlighted in the previous draft of the paper but required greater clarity in their presentation. |
| DISCUSSION. (No line numbers in this part) 37.Paragraph 2. Most of the ideas presented in this paragraph pertaining to relevance needs to be elaborated on. For example, the concept that since there is a large quantity of MS research then it is perceived to be more relevant? What is the basis for saying this? Similarly the sentence/concept related to wider health care system and funding model of direct care need explanation and elaboration to better understand how this is related to decrease in Relevance domain of physios in the workplace. Finally the last concept there on competing demands of improving clinical skills…etc. needs further explanation on how it affected perception of relevance for EBP. | Paragraph 2 in the discussion section has been re-written (see Appendix below) to address the reviewer’s comments, with a further detail provided regarding the aspects related to relevance. |
| 38.Paragraph 3. On reduced confidence. Improving sentence construction of this could help in delivering the message clearly. Eg. The overall decline in the Confidence domain of the participants may have been influenced by their reported barriers to use EBP. These barriers would lead to less frequent practice of looking up evidence and thus possibly decrease confidence. Similarly, McEvoy’s study showed that…. You mentioned that McEvoy [13] study did not use qualitative data to explore…how then do you think your data is better or different from this study and how does this current study better explain the decrease in confidence? | Paragraph 3 has been re-written to address the reviewer’s comments (see Appendix below). |
| 39.Paragraph 4. on workplace culture. Shifting to an active voice could better deliver the message. Ex. Results of this study indicate that a workplace culture that values research evidence strongly supports EBP use and helps in maintaining/improving ? confidence. Some of the practices that cultivate this culture include….(state here). | Paragraph 4, (now paragraph 5) of the Discussion, has been re-written to address the reviewer’s comments (see Appendix below). |
| 40.Paragraph 5. Transition to workplace Is this all about PEAK? Maybe just give a summary of what this is about and then provide your own insight about this? Maybe there are other options? | Paragraph 5, (now paragraph 6) of the Discussion, has been re-written to address the reviewer’s comments (see Appendix below). |
| 41.Paragraph 6. Recommendations. It might be helpful to organize your thoughts for this…recommendations in education and recommendation in practice? (you referred to this in your introduction, line 77). Did the data lead you to believe that improvement in EBP education in entry-level could help maintain the Profile domain scores? You mentioned that there are several recommendations to be considered but actually only mentioned the ‘re-fresh’ training. It might help if you outline the recommendations and discuss each. | Paragraph 6, (now paragraph 7) of the Discussion, has been re-written to address the reviewer’s comments (see Appendix below). |
| 42.Paragraph 7. Limitations. No need to state strengths, just disclose limitations. | The sentence relating to strengths at the beginning of paragraph 7 (now paragraph 8) in the Discussion has been removed. |
| I question your second limitation; "of using descriptive thematic analysis and not allowing for rich in-depth exploration of the findings”. Your purpose for collecting qualitative data is to gain a deeper and broader understanding (line 94-95). You further described the use of an interpretive paradigm to allows rich description of lived experiences. Your description of qualitative data analysis emphasized an iterative approach to make sure that nothing is left undiscussed and undecided. Yet you say that this is not a rich in-depth exploration of the findings? This limitation does not follow through with your intention in using qualitative analysis | We acknowledge it was an error in identifying our thematic approach as a limitation. Please see our response to point 6. |
| 43.Your third limitation also puts to question the validity of the quantitative data. What is the objective of your study? Did you aim to determine how these factors influence the data? (I mentioned that you should also include an objective for your quantitative arm in the introduction). | The specific aims of the study outlined in the introduction section (lines 83-87)of the manuscript have been revised to explicitly state the objective of both the quantitative and qualitative arms of the study. This section now reads:  “Therefore, the objective of this study was to determine what is influencing the use of EBP by physiotherapists during their first year in the workforce. The specific aims of the study were to quantitatively examine overall changes in domain scores associated with EBP knowledge, attitudes and behaviours, and to qualitatively explore the experience of using EBP in a cohort of physiotherapists after a year in the workforce.”  In addition, the section of the Discussion regarding the final limitation regarding potential confounders such as place of employment and locations has been amended and reads:  “Secondly, it is possible that there were confounders such as place of employment, or rural, metropolitan and remote locations. As the primary aim of the quantitative arm of this study was to assess the change in domain scores after a year in the workforce, these variables were not assessed or included in the analyses. This is a possible area for future research.” |
| CONCLUSION. 44.This part should go back to the research questions/ or research objective as stated. One year into working, physiotherapists showed decline in all EBP profile domains, most signficantly for…. In the first year of work, the participants experience of EBP revolved around the themes of… | The conclusion has been re-structured, to relate to the study aims and research question. The conclusion previously read:  “In conclusion, domains related to EBP knowledge, attitudes and behaviour in physiotherapists decline after a year in the workplace, with significantly reduced confidence and perception of the relevance of EBP. A subtle interplay of features related to workplace culture, competing demands to develop clinical skills, communication skills and expectations of patients, together with availability of resources and time, may impact early graduates’ perceptions of EBP. Role models in and outside of the work environment who immersed themselves in evidence discussion and experience were identified by early graduates as inspiring.”  The conclusion now reads:  “In conclusion, one year into working, physiotherapists showed a decline in all EBP profile domains, most significantly for confidence and the perceived relevance of EBP. In the first year of work, a subtle interplay of features related to workplace culture, competing demands to develop clinical skills, internal and external motivators to use EBP and expectations of patients, together with availability of resources and time, may impact early graduates’ perceptions of EBP. Role models in and outside of the work environment who immersed themselves in evidence-based discussion and experience were identified by early graduates as inspiring.” |
| **Reviewer #2**: | Thank you for your considered review of our paper. We have responded in the table below to each point you have raised. |
| 44.It would however, be beneficial to identify for the reader whether a convergent or sequential (explanatory/exploratory) design was taken for this mixed methods approach. This would help to situate the qualitative work better in the study design. | This point was also raised by the other reviewer (point 3) and highlighted our lack of detail in this section of our paper. We have now added information and a reference to address this.  Page 5/6, lines 94-99 previously read,  “This mixed-methods study used quantitative and qualitative methods to collect information from participants. Quantitative data were collected with two questionnaires at two timepoints (graduation and after one year in the workforce). Qualitative data were collected with individual interviews at one timepoint (after one year in the workforce) to gain a deeper and broader understanding of graduates’ first year of work and EBP experience. “  This now reads, page 6, lines 104-113:  “Quantitative and qualitative procedures of the study were conducted independently of each other in a convergent mixed methods design [17]. The timepoint 1 quantitative data was collected on participants’ completion of the final EBP course in their final year of the Physiotherapy Program. The timepoint 2 quantitative data was collected after participants had been in the workforce for up to 1 year (September-November 2014). The qualitative interviews were conducted in the immediate period after the timepoint 2 quantitative data was collected (November 2014). The interview questions were independent of the quantitative results and not all participants who completed the survey, consented to the interviews. The quantitative and qualitative data were analysed separately then merged in the interpretation and reporting of results.” |
| 45.While the qualitative methodology describes a staged inductive approach to thematic analysis, the descriptive approach used to report results leads to many (maybe too many) sub-themes which tended to match back to the questions asked. To this end it would be helpful to know more about the design of the question schedule and whether the quantitative data findings informed this process. Overall descriptive thematic analysis would not be my preferred choice as the results generating 9 themes with multiple sub-themes made for lengthy and difficult reading with limited meaningful synthesis to take away. | Thank you for your feedback.  The specific aim of each arm of the study is now stated at the end of the Background section on page 5, lines 83-87:  “Therefore, the objective of this study was to determine what is influencing the use of EBP by physiotherapists during their first year in the workforce. The specific aims of the study were to quantitatively examine overall changes in domain scores associated with EBP knowledge, attitudes and behaviours, and to qualitatively explore the experience of using EBP in a cohort of physiotherapists after a year in the workforce.”  The design of the interview schedule was to address the qualitative aim stated above and was not informed by the quantitative data findings. The ‘Qualitative data collection’ section on page 8, lines 168-170, has been revised to read,  “A semi-structured interview guide was developed by the research team independent of the quantitative data findings  We acknowledge your critique of the number of sub-themes and limited synthesis in the qualitative data findings. The points raised by both reviewers prompted us to return to the analysis for further iterative work across codes and categories to define themes that are meaningful to the research aim of how the participants experienced using EBP in their first year in the workforce. A Braun and Clarke (2012) reference [24] informing this thematic analysis process has been added to the Methods ‘Data management and analysis’ section.  The reporting of the qualitative results has been revised from page 14 and uses narrative reporting of seven key and meaningful themes with illustrative quotes only in the text as suggested. The seven key themes describe the key findings of the qualitative analysis, which had already been highlighted in the previous draft of the paper but required greater clarity in their presentation. |
| 46.In the qualitative results table some participants are labelled xx and this needs to be addressed to assure fidelity of data. | Table 2 has been removed from the paper. The correct participant number has been assigned to the illustrative quote now presented under Theme 3 in results. |
| 45.With respect to the quantitative study, more detail relating to the data (sd etc) and sources of this data used in the power calculation would be helpful for readers. | A reference has been added relating to the source of data used for the power calculation.  This now reads (as reported in point 18 above):  “A minimum number of 34 participants was needed for the quantitative data set in order to obtain 80% power for a two-tailed distribution, with alpha set at 0.05 and a medium effect size (d=0.5) derived from the study of McEvoy et al [13].” |
| 47.The authors highlight that the EBP questionnaire which uses Likert scales would be treated as interval data, however the same information and justification for parametric testing of the K-REC instrument is not provided. Similarly in the reported results, it would be better to address the K-REC results separately as it is a different construct with different psychometric properties to those in the EBP profile questionnaire. | The K-REC data was analysed separately and is presented separately in Table 1.  The K-REC data is presented in (now) Figure 2 as a domain that is considered to assess actual rather than reported knowledge. |
| 48.The discussion section adopted a very narrow approach to the findings, lacking higher level discourse which was disappointing. In fact only two new references to the literature were introduced at this stage. Acknowledging the paucity of studies directly related to this research question, I would have expected to see a wider discussion in relation to the qualitative literature addressing transitioning to professional practice following graduation and similarities with the findings in this study. | The discussion has been broadened and a wider discussion has been included with comparison of qualitative literature relating to transitioning to professional practice. |
| 49.Similarly, I would have expected reference to stages of learning as novice, advance beginner, proficient, competent, and finally expert where ones does see greater confidence in novices than beginner/proficient learners as recognition of contextual factors and greater insight into deficits in knowledge become more apparent. | The authors agree that reference to the different stages of learning will strengthen the study. An additional paragraph (now paragraph 4) has been added to the Discussion to address stages of learning:  “A decline in confidence in *clinical practice* in the first year of practice in physiotherapy has also been reported [26], with a greater ‘outward’ focus and confidence in the second year of practice. When their EBP skills are viewed in the context of the five stages of learning, graduates in the current study may have moved through novice and advanced beginner levels during EBP training across the physiotherapy program, and enter the workforce at a competent level [27]. Coaching and a balance of supervision and autonomy, with encouragement to self-reflect and justify decisions may have supported achievement of this level of EBP skills [28]. To reach the fourth level of proficiency in EBP skill there needs to be support in decision-making and self-reflection in relation to more complex experiences to build confidence [28]. This level, which is characterised by taking responsibility for one’s decisions, identifying opportunities to teach, and being responsible to others, may be stalled in the first year of working with the potential to re-emerge after two years as a working physiotherapist.” |
| 50.One minor consideration that was confusing was that in the intro pg4 line 65 they cite McEvoy et al [13] as providing workplace transition qualitative data and in the discussion paragraph 3 they cite the same study as providing longitudinal data that lacks qualitative insights. | Thank you for pointing this out. This was an error. McEvoy et al [13] provided quantitative data only, and no qualitative data. This error has been corrected. The text (lines 65-67) now reads:  “McEvoy et al. [13] also provided quantitative data on changes in EBP knowledge, attitudes and behaviour after transitioning into the workplace for one and two years. However, there was no further exploration of what may influence these changes.” |

**APPENDIX**

**Fig 2 (previously Fig 1)**

**Paragraphs in the discussion have been re-written.**

Below are previous and new paragraphs in the Discussion

**Paragraph 2 previously read:**

Relevance of EBP in clinical practice showed an overall decline after one year in the workforce but the qualitative findings suggest this varied according to area of clinical practice. Participants reported EBP, having greatest relevance to the management of musculoskeletal conditions compared to their experience in areas of aged care, paediatrics and inpatients All clinical areas in physiotherapy are actively researched and have research evidence available to clinicians. It is possible that the large quantity of musculoskeletal research in physiotherapy conference programs and many journals is influencing the perception of relevance, rather than the quality of the research available. It may also be that the wider health care system impacts on physiotherapists practice and ability to use EBP eg where funding models direct care. Furthermore, the perceived relevance of EBP may also be influenced by competing demands of improving clinical skills, building rapport and learning from experienced colleagues and the perceived lack of EBP expectation from many patients.

**Paragraph 2 now reads:**

Relevance of EBP in clinical practice showed an overall decline after one year in the workforce but the qualitative findings suggest that this varied according to area of clinical practice. Participants reported that EBP had greatest relevance in the management of musculoskeletal conditions where presentations were varied and progressed through stages of rehabilitation. There was a perception of greater autonomy in management and integration of best research evidence in clinical reasoning. By comparison, participants felt the value and use of EBP in other clinical areas was limited by little or slow changes in practice. All clinical areas in physiotherapy are actively researched and have research evidence available to clinicians. It is possible that the large quantity of musculoskeletal research in physiotherapy conference programs and many journals influenced the perception of relevance, rather than the quantity or quality of the research available. It may also be that wide health care systems impact on physiotherapists’ practice and ability to use EBP e.g. where funding models direct care. Furthermore, the perceived relevance of EBP appeared to be influenced by competing demands for early career physiotherapists to learn from experienced colleagues and improve clinical skills. Relevance may also have been impacted by the perceived lack of expectation regarding EBP from patients. However, when evidence is explained, many patients appreciate the decision-making underpinning treatment choices and communicating evidence needs to be recognised by practitioners as a professional obligation relevant to the communication requirements of informed consent [25].

**Paragraph 3 previously read:**

The overall decline in confidence to use EBP were reportedly influenced by reduced resources, time to access and interpret evidence and reduced opportunities to use EBP. Less support may also have contributed to reduced motivation and therefore less practice may have led to reduced confidence. A decline in confidence after working for one year reported previously in a longitudinal study by McEvoy et al. [13], was followed by improved confidence in the second working year. The absence of qualitative data in that study limited exploration of possible contextual and environmental influences, but it was hypothesised that early graduates may need an initial period to establish workplace relationships and clinical routines before their focus and confidence returns to EBP.

**Paragraph 3 now reads:**

The overall decline in the Confidence domain identified in this current study may have been influenced by participants reported difficulties in accessing the time and database evidence for practicing EBP. Participants reported retaining skills for searching databases, but were generally less critical of research with poorer ability to interpret the statistical measures and the quality of the research. A lack of consistent practice and a loss of knowledge in skills for accessing, appraising and integrating best research evidence were cited as contributors to having less confidence. Interestingly, following an initial decline after a year of working, McEvoy et al [13] reported improved confidence scores after two years of working. The authors hypothesised that early graduates may need an initial period to establish workplace relationships and clinical routines before their focus and confidence returns to EBP.

**Old paragraph 4, now paragraph 5, on workplace culture previously read:**

A workplace culture where there is discussion of research evidence, sharing of articles, support and encouragement to attend evidence-based courses and report on findings emerged as pivotal for supporting the use of EBP in the workplace and perceived confidence in EBP skills of new graduates. Contributing also to the EBP culture, were availability of synthesised research evidence compiled by staff and free database access with an expectation of using it. Role models both inside and out of the workplace who championed and practiced EBP and undertook research were inspiring to new graduates. In interviews, graduates identified employers who challenged them to present supporting evidence for managements, colleagues whom they observed reading articles, presenters commenting on the appraised quality of studies and others who engaged in research themselves, as examples of role models (Table 2).

**New paragraph 5 now reads:**

The results of this study indicate that a workplace culture that values research evidence strongly supports EBP use and helps in maintaining confidence and supporting EBP practice in the workplace. Some of the practices that cultivate this culture can be implemented in all workplaces, including ongoing discussions of research evidence, sharing of journal articles, regular in-service education, and the support and encouragement by supervisors to make evidence-based decisions. Role models within and outside the workplace who championed and practiced EBP were inspiring to new graduates and can be considered key motivators to using EBP in the first year of work.

**Old paragraph 5, now paragraph 6, (Transition to workplace) previously read:**

There are no known previous studies that have evaluated the changes in EBP knowledge, attitudes and behaviours in this transitional period from graduation to a year in the workplace. Several studies have suggested that EBP training should be integrated across the professional continuum rather than constrained to entry-level programs prior to graduation and registration [23-24]. Encouraging workplace-support and mentoring, Tilson and her team [8-10] developed a program (PEAK: the Physical therapist-driven Education for Actionable Knowledge translation program) to support integration of research evidence into clinical decision-making. Opportunities are provided for expert and peer support to explore new skills, and small group learning with an experienced EBP expert, librarian and with peers, to encourage greater confidence and sustained research evidence integration into practice [9]. The PEAK program improved self-reported EBP confidence and behaviours. However, at six-months there was low objectively-assessed adherence (based on case note reporting) to an agreed list of evidence-based behaviours (managements) developed by participants [10]. To sustain implementation of knowledge, Tilson et al. [10] proposed incorporating greater organisational support with more monitoring, feedback and problem-solving.

**New paragraph 6 now reads:**

There are no known previous studies that have evaluated the changes in EBP knowledge, attitudes and behaviours in this transitional period from graduation to a year in the workplace. Several studies have suggested that EBP training should be integrated across the professional continuum rather than constrained to entry-level programs prior to graduation and registration [29-30]. Relating to EBP in psychology, Leffler et al [29] proposed individualised training and active strategies that are supported and have measurable outcomes of learning and effects. Examples of these approaches during training, during internship and for clinicians were provided. While not always available, internship is closest to the first year of practice. Leffler et al [29] proposed a dedicated period to train psychologists to integrate clinical research and practice, by the collaborative development of projects under the supervision of faculty members who engage in clinical research. Tilson and colleagues [8-10] developed a program *for clinicians* to support integration of research evidence into clinical decision-making. In the Physical therapist-driven Education for Actionable Knowledge translation program (PEAK), opportunities are provided for expert and peer support to explore new skills, using small group learning with an experienced EBP expert, librarian and with peers [9]. The PEAK program improved short term self-reported EBP confidence and behaviours, but this was not sustained at six months [10]. Tilson et al [9, 10] did not report on whether participants had exposure to EBP during physiotherapy training. McEvoy et al [12] found significant improvement in self-reported EBP knowledge, attitudes and behaviours prior to graduation, after exposure to EBP courses. These improvements in the same cohort after graduation were not sustained, as reported in the current study. Lessons may be learned from Tilson et al [9, 10], to further build on a strong EBP foundation gained prior to graduation.

**Old paragraph 6, now paragraph 7, (Recommendations) previously read:**

Reflecting on this related research and the current study findings of changes in EBP confidence and the impact of workplace culture and role models, several recommendations could be considered. To continue to maintain and grow EBP in the profession there appears to be a need to ‘re-fresh’ training in the 5 steps of EBP (ask, acquire, appraise, apply and assess) in the second working year together with discussion about where and how EBP is most useful in the workplace. This could be in the context of new graduates’ workplace experience, the processes associated with EBP, the accessibility to databases and the necessary resources. If these early graduates of today, are to be the role models for tomorrow’s graduates, then investing in additional EBP support to develop leadership may pay dividends in enhancing the aspiring evidence-based practice health professionals.

**New paragraph 7 now reads:**

Recommendations from this research relate to clinical practice and education. A practice recommendation for the future may be to incorporate greater organisational support, with more monitoring, feedback and problem-solving offered in the transition period using EBP experts, librarians and peers. In addition, building stronger links between workplaces and physiotherapy training institutions may allow the mutual strengths of both to be better utilised. For example, students often have research and analysis skills to share, but need consolidation of clinical skills that can be provided by experienced clinicians. Together there can be integration of clinical and research evidence along with evidence from the patient, to provide authentic EBP.

Education recommendations may be to target statistics training focussing on *application* of statistics, to build confidence in this area. This should not be at the expense of continued development of all EBP skills, as entry-level education resulted in significant improvements in all domains [12]. A further recommendation may be to better prepare students for the challenges of the transition period where workplaces may provide limited support, and to encourage new graduates to seek role-models and opportunities to maintain EBP skills.
